# Cancer Cell Line Inhibition by Osmotic Pump-administered Xylitol in a Syngeneic Mouse Model

**DOI:** 10.21203/rs.3.rs-3977059/v1

**Published:** 2024-04-02

**Authors:** Mark Cannon, Elizabeth Dempsey, Ashlee Cosantino, Navdeep Chandel, Nayereh Ghoreishi-Haack

**Affiliations:** Robert H Lurie Comprehensive Cancer Center -Northwestern University; Northwestern University; Northwestern University; Robert H Lurie Comprehensive Cancer Center -Northwestern University; Northwestern University

**Keywords:** Alzet osmotic minipumps, malignant melanoma, xylitol, syngeneic mouse model

## Abstract

**Background:**

This study aimed to evaluate the effects of continuous administration of xylitol (a commonly used dental prebiotic) via a subcutaneous osmotic minipump in a B16F10 syngeneic mouse model.

**Methods:**

The B16F10 syngeneic model consisted of 6–8-week-old C57BL/6 male mice subcutaneously injected with five × 10^5^ B16F10 cells suspended in 100 μl PBS in the right flank. The mice were randomly assigned to two groups: Group 1 was the treatment group with 10% xylitol-loaded pumps (n=10), while Group 2 was the control group with saline-loaded pumps (n=10). Alzet minipumps were implanted subcutaneously in the left flank of B16F10-injected mice once more than 50% of all mice developed palpable tumors. After pump implantation surgery, the mice were monitored daily and weighed 2–3x/week. Tumor sizes were measured with calipers 2–3x/week, and all mice were euthanized when their tumors became too large (20 mm on any axis or 2,000 mm^3^). The excised tumors were weighed and cut in half, with one half sent for histology and the other for metabolomic analysis.

**Results:**

The xylitol-treated group survived substantially longer than the control group. The tumor size was reduced by approximately 35% by volume. Histological sections of xylitol treat mice suggested reduced infiltration and angiogenesis, which is consistent with previous studies. The metabolomic analysis demonstrates that xylitol reduces the tumor production of histamine, NADP+, ATP, and glutathione from the tumor, thereby improving the host immune response with ROS reactive oxygen species.

**Conclusions:**

The results of this study suggest that xylitol has potential as an adjunct to oncological treatment and is being further investigated in comparison to monoclonal antibody therapy (Opdualag).

## Background

Xylitol is a prebiotic polyol long utilized for preventive care in dentistry and is considered a natural and generally regarded as a safe food additive or supplement [1, 2]. It has long been used as a substitute for ordinary table sugar owing to its sweetness and taste profile, which are similar to sucrose [3]. In 1975, Makinen et al. first reported that xylitol significantly reduced dental caries by inhibiting the growth of *Streptococcus mutans* [4]. Since then, clinical studies have demonstrated that xylitol products decrease the oral microbiome levels of *S. mutans*, the amount of plaque, and the incidence of dental caries in children [5–7]. Total or partial substitution of sucrose with xylitol in the human diet reportedly results in more than an 85% reduction in the incidence of dental caries [8]. According to Mäkinen et al., most *S. mutans* strains transport xylitol into the cell via the phosphotransferase system, where it is then phosphorylated to xylitol-5-phosphate and expelled from the cell [9, 10]. This energy-consuming pathway is thought to inhibit *S. mutans* [11]. Erythritol has been reported by Mäkinen et al. to prevent dental caries; however, few studies regarding this have been published [12, 13]. Numerous studies have demonstrated the efficacy of xylitol in preventing caries and inhibiting periodontal pathogens. In addition, xylitol provides balance and maintains a healthy microbiome, beginning with the oral gateway microbiome, which supports innate immunity and disease resistance [14–17].

The microbiome’s influence on cancer development and treatment has now been recognized [18]. Research on complex microbial communities and the mechanisms through which microbiota influences cancer prevention, carcinogenesis, and anticancer therapy have significantly increased. Researchers are considering the urgent development of next-generation prebiotics and probiotics designed to target specific diseases [19]. Healthcare professionals will soon supplement effective prebiotics, probiotics, and derived postbiotics to prevent and treat disease [20]. The hallmarks of cancer are immune elimination and escape, both of which can be partly bacteria-dependent, shaping immunity by mediating host immunomodulation [21]. In addition, host immunity regulates the microbiome by altering bacteria-associated signaling to influence tumor surveillance [22]. Cancer immunotherapy, including immune checkpoint blockade, appears to have heterogeneous therapeutic effects in different individuals, which have been partially attributed to the microbiota [23]. Personalized medicine requires a better understanding of the microbiota and their interaction with cancer cells. The manipulation of the gut microbiota to improve cancer therapeutic responses may be indispensable for future cancer treatment [24].

Xylitol inhibits cancer cells when dietarily and systemically administered [25–27]. Because xylitol is utilized by healthy human cells and exhibits almost no side effects, it is potentially a safe supplement to inhibit cancer cell proliferation [28, 29]. Xylitol also decreased tumor vascularization by inhibiting angiogenesis. Increased vascularization supports tumor growth and possibly cancer metastasis [30]. Xylitol is natural, and humans are reported to produce approximately 15 g of xylitol per day in the liver [31]. Xylitol is converted by mitochondrial xylitol dehydrogenase into a precursor of the tricarboxylic acid cycle. Xylitol dehydrogenase on cristae metabolizes xylitol to xylulose, which converts NADP to NADPH [32]. Many plants naturally have substantial amounts of xylitol present, such as blueberries, strawberries, plums, cauliflower, and oatmeal [33], in addition to the production of xylitol by the human liver. Interestingly, there is some overlap between the list of xylitol-containing foods and the American Heart Association’s list of “heart-healthy” foods. Xylitol has been suggested to prevent diabetes and as an anti-inflammatory agent [34, 35].

Previously published research on animal models has reported positive results in inhibiting cancer cell lines and cancer xenografts with xylitol supplementation [36]. Combination treatments with olive oil phenolic compounds and xylitol have also been reported to be potentially beneficial [37]. The first phase of cancer research, which involves looking for potential therapeutic agents, typically involves animal models [38,39]. Therefore, the present study used a syngeneic malignant melanoma mouse model to evaluate the inhibitory efficacy of xylitol solution delivered via Alzet osmotic minipumps.

## Materials and Methods

**Figure 1:**
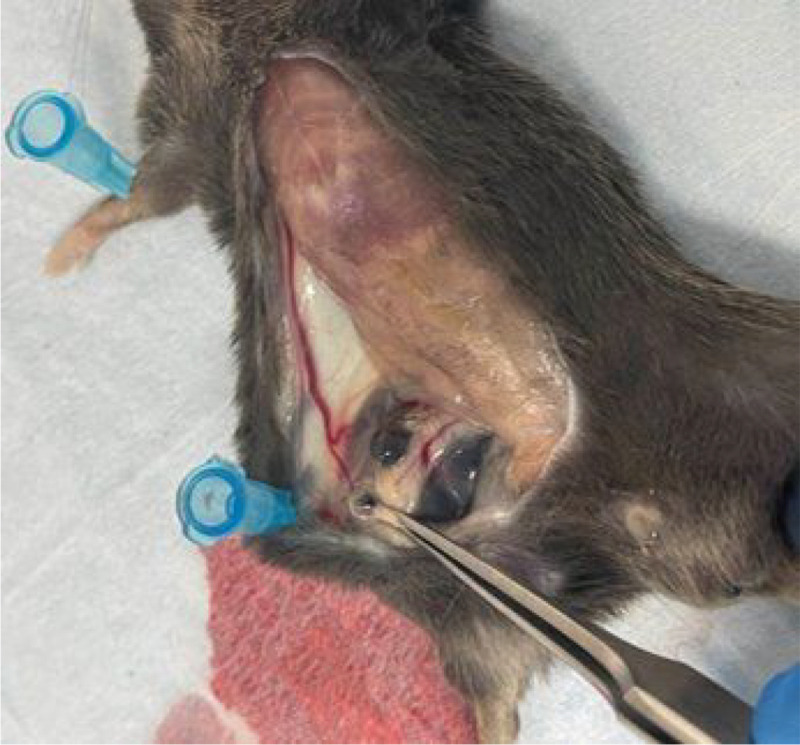
Excision of the Tumor and Lymph Nodes in animal model. The tumors were sectioned, with half sent for histological analysis and the other half sent for metabolomic analysis. Lymph nodes were submitted for histological examination to determine the level of metastasis.

B16F10 Syngeneic Model consisted of 6–8-week-old C57BL/6 male mice subcutaneously injected with 5 × 10^5^ B16F10 cells suspended in 100 μl PBS into the right flank. Mice were monitored for tumor development and randomized according to tumor volume and body weight for implantation surgery. The mice were randomly assigned to two groups: Group 1 was the treatment group with 10% xylitol-loaded minipumps (n = 10), while Group 2 was the control group with saline-loaded minipumps (n = 10). Alzet minipumps were implanted in B16F10 injected mice once more than 50% of the mice developed palpable tumors. Alzet osmotic pumps (Model 2004) were loaded with either a 10% xylitol solution in saline or saline vehicle controls. The pumps were primed at 37°C for 48 hours before the implantation surgery. All minipump surgeries had to be completed in one day, and the minipumps were loaded >40 hours before surgery. The minipumps should have released xylitol for 28 days. Twenty mice underwent surgery to implant Alzet minipumps and were group-housed before and after surgery. Each mouse was placed under isoflurane anesthesia (~ 3%) and then given preoperative analgesia (SQ: 20 mg/kg meloxicam, line block of 2 mg/kg 0.1% bupivacaine). Under sterile conditions, an incision of approximately 1 cm was made in the lower left flank. Pumps were placed subcutaneously in the lower left flank, and for most mice, the surgical incision was caudal to the pump. The incisions were closed using 2–3 wound clips. The mice were monitored 2x per day for the first 48 hours post-operation. After minipump implantation surgery, the mice were monitored and weighed 2–3x/week. Tumor sizes were measured with calipers 2–3x/week, and all mice were euthanized when their tumors were too large (20 mm on any axis or 2,000 mm^3^). The excised tumors (Fig. 1.) were weighed and cut in half, with one half sent for histology and the other for metabolomic analysis. In addition to tumor tissue, draining lymph nodes were also collected and sent for histological analysis.

Rodent CO2 Euthanasia was performed according to IACUC protocol. Animals were not combined from different cages, and when euthanizing an entire cage, the animals remained in their original housing. The maximum number of mice per cage was five mice, and the CO2 flow rate per mouse cage was 3 L/min until one minute after breathing stopped. Euthanasia was confirmed by cervical dislocation.

## Results

**Figure 2:**
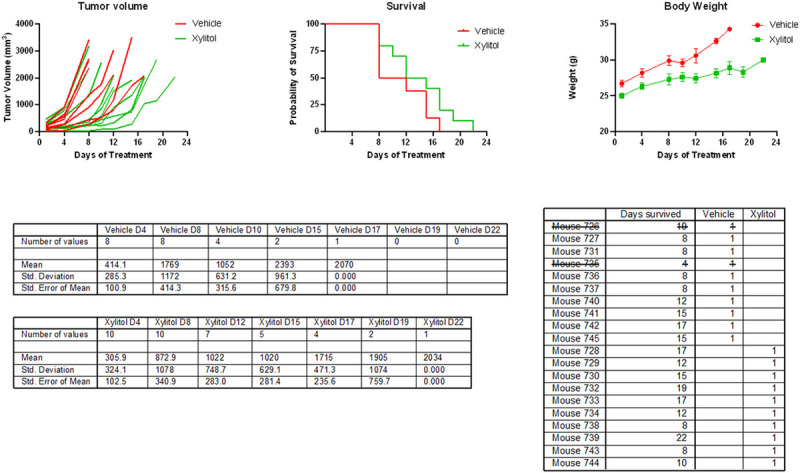
Score Plots with Tumor volume, Survival of animal models, and Body Weight graphs. The Xylitol group demonstrated increased survival, with less tumor volume and less weight gain due to tumor growth. Note: Mouse #735 was excluded from calculations because its tumor size was too large at the time of randomization, and mouse #726 was excluded due to poor tumor growth.

**Figure 3:**
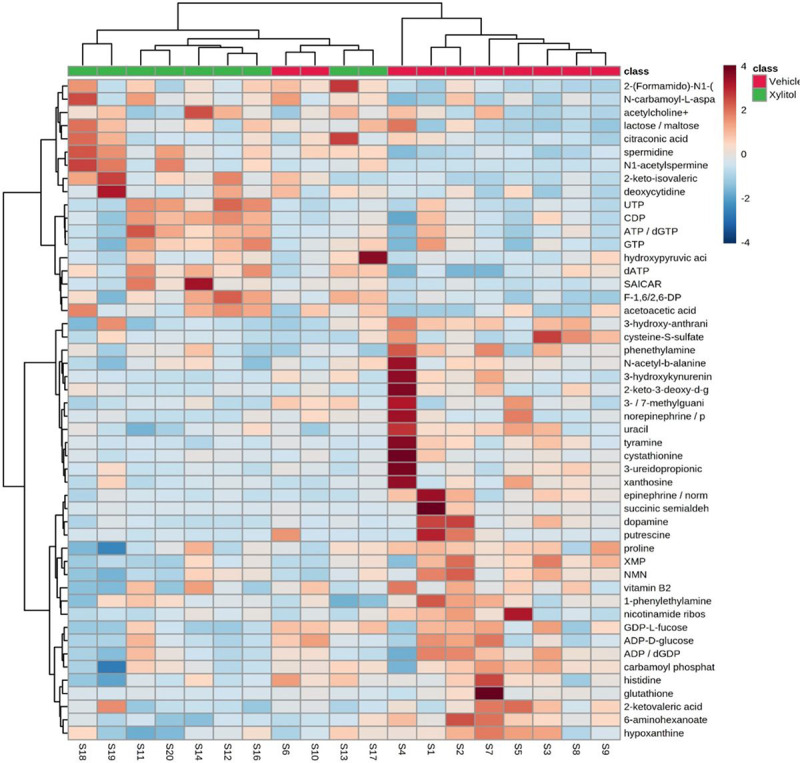
Metabolomics chart of the tumor cells comparing xylitol to vehicle-treated syngeneic mice models. Significant differences exist between the experimental (xylitol) and vehicle-treated (control) groups. Note increases in the proline, NMN, and XMP with the vehicle group. The xylitol group had increases in UTP, CDP, ATP, and GTP. In addition, the xylitol group showed decreases in the phosphocreatinase, pyruvate, citrate, and glutathione present.

The survival of the xylitol-treated group was substantially longer than that of the control group. The tumor size was reduced by approximately 35% by volume (See Fig. 2.). The weight gain in the xylitol groups was also less than in the vehicle group, possibly related to the decreased tumor size. Histological sections suggested reduced infiltration and angiogenesis, which is consistent with previous studies. Metabolomic analysis (Fig. 3.) demonstrated that xylitol reduced the tumor production of histamine, NADP+, ATP and glutathione, possibly improving the host’s immune response to tumor cells by reactive oxygen species production. Significant differences existed between the experimental (xylitol) and vehicle-treated (control) groups. Note the proline, NMN, and XMP increase with the vehicle group. In addition, the xylitol group showed decreases in the phosphocreatinase, pyruvate, citrate, and glutathione present compared to the control group.

## Discussion

The present study was part of a series of syngeneic models to evaluate the effects of xylitol on tumor cell proliferation and discern any improvement in the survival of the subject mice. The use of Alzet pumps was suggested due to the inadequacy of the intratumoral injection protocol. Intratumor injections resulted in stromal disintegration and subsequent xylitol leakage. In addition, the 20% solution may have been too concentrated, creating local areas of crystallization and preventing proper dissemination. Alzet pumps could exhibit crystal deposition above a 10% solution, and occasionally, there would be an area of poor wound healing, perhaps reducing the potential for increased survivability. However, metabolomics revealed a significant change in metabolites within the tumor when xylitol was administered instead of saline control. This finding is consistent with that of our previous research and other published scientific articles [26, 27, and 29]. The metabolomic analysis demonstrated that xylitol reduced the production of histamine, NADP+, ATP and glutathione from the tumor, thereby improving the host immune response to reactive oxygen species produced by innate immune cells.

Possibly, osmotic minipumps may be inserted into large tumors to deliver a constant supply of xylitol that inhibits the growth of certain cancer cell lines without any side effects. Alternatively, xylitol could be systemically administered during the treatment phase along with standard-of-care therapy, followed by the use of dietary xylitol supplements to prevent recurrence. The consumption of high-sucrose foods should be discouraged, and that of xylitol-containing products, fruits, and vegetables should be encouraged with oncology patients. Although controversial, sucrose and fructose substitutes are safe and widely available. Multiple studies have demonstrated Xylitol inhibits certain cancer cell lines and is readily incorporated into the diet, replacing sucrose and fructose. The survival times of xylitol-treated mice were up to 40% higher than that of control mice. Large clinical trials should be considered to evaluate the benefits of xylitol in cancer cell lines and syngeneic mouse models. No obvious side effects of the xylitol treatment were noted in the experimental group.

## Conclusions

The results of this study suggest that xylitol has the potential to be an adjunct to oncological treatment and is being further investigated by comparing its effectiveness to a monoclonal antibody (Opdualag). An osmotic minipump (Alzet) provided constant systemic exposure to 10% xylitol solution, which reduced the growth of malignant melanoma tumors and increased the survivability of xylitol-treated mice.

## Data Availability

not publicly available at this time.
